# Development and Validation of a Double-Sensor Hump Calibration Method for Articulated Vehicle Model Identification

**DOI:** 10.3390/s23249691

**Published:** 2023-12-07

**Authors:** Yuhang Wu, Yuanqi Li

**Affiliations:** 1 Department of Structural Engineering, Tongji University, Shanghai 200092, China; 1810187@tongji.edu.cn; 2 State Key Laboratory of Disaster Reduction in Civil Engineering, Tongji University, Shanghai 200092, China

**Keywords:** simulations, articulated vehicle models, double-sensor hump calibration method, sensor layout optimization, validation, experimental measurements, laden vehicles

## Abstract

The realistic simulation of the dynamic responses of a moving articulated vehicle has attracted considerable attention in various disciplines, with the identification of the vehicle model being the prerequisite. To this end, a double-sensor hump calibration method (DHCM) was developed to identify both unladen and laden vehicle models, consisting of a sensor layout optimization step and a system identification step. The first step was to optimize the number and position of sensors via parameter sensitivity analysis; the second was to inversely identify the vehicle system based on sensor responses. For comparison, the DHCM and the existing single-sensor hump calibration method (SHCM) were used to calibrate a small-sized vehicle model and a multi-axle articulated vehicle model. Vertical accelerations of the vehicle models were then simulated and characterized by power spectral densities (PSDs). Validation against experimental measurements indicated that the PSDs of the models identified with the DHCM matched the measured PSDs better than those of the SHCM, i.e., the DHCM-identified model accurately simulated the dynamic response of an articulated vehicle with relative errors below 16% in the low-frequency range. Therefore, the DHCM could identify models of small-sized vehicles and multi-axle articulated vehicles, while the SHCM was only suitable for the former.

## 1. Introduction

A pitch-plane vehicle model with hinges connecting the individual articulated parts describes the bouncing and pitching motions of a multi-axle articulated vehicle. Since the dynamic responses such as accelerations and forces associated with the above motions can be captured based on the known equation of motion of the vehicle model and the input road profile conditions [1,2,3], calibration of the parameters of a multi-axle articulated vehicle model is a crucial requirement to obtain realistic dynamic responses of the articulated vehicle. Recently, much attention has been paid to simulating the dynamic responses of a moving articulated vehicle involving multiple disciplines. For example, in the food industry, dynamic loading, primarily referring to the vertical accelerations of multi-axle articulated vehicles (e.g., tractor-semitrailers), is the leading cause of damage to transported food products. Simulation of dynamic loading helps to evaluate such mechanical damage [4,5,6,7,8]. In addition, with the development of prefabricated construction technology, attention must be paid to transporting heavy objects, such as building modules [9], a long cuboid structure that needs to be transported from the factory to the construction site. Using dynamic loading (also termed transportation loading [10]) as input conditions, FEM-based vibration analysis [11,12] has predicted the failure of building modules due to fatigue. Moreover, vehicle–bridge interaction (VBI) dynamics research considers a moving vehicle as a vibration source that excites a bridge structure. The dynamic characteristics of the bridge are determined based on the generated tire forces of the vehicle [13]. Compared with sedan vehicles, which are limited to determining the dynamic properties of short-span bridges [14,15,16], the excitations generated by articulated vehicles have been used to determine the properties of short, medium, and long-span (mostly long-span) bridges [17,18,19,20,21,22]. The above representative research areas demonstrate the innovation, applicability, and importance of articulated vehicle dynamic responses, including vertical accelerations and tire forces, in multidisciplinary research. However, regardless of the location or category of vehicle dynamic responses, realistic simulations can only be achieved using the equations of motion of identified multi-axle articulated vehicle models.

Indeed, simulations of dynamic responses can be performed as realistically as possible, provided that the parameters of the model of a multi-axle articulated vehicle are calibrated. Therefore, the following literature review focuses on the calibration methods of multi-axle vehicle models, which are classified into the conventional method, immature inverse method, and hump calibration method. Traditionally, obtaining vehicle parameters is time-consuming due to requiring information from vehicle manufacturers or performing complicated laboratory tests. For example, Davis [23] conducted a series of tests, including on-road, step, and pipe tests, to determine the suspension parameters of heavy vehicle models. An alternative to the conventional method is the inverse method. Theoretically, the vehicle calibration process can be considered as an inverse problem, which is generally positioned as the problem of determining the parameters of a system from its input–output correspondence [24]. In practice, Rozyn et al. [25] used measurements of the response of the sprung mass when a vehicle was driven over an unknown road to determine the parameters of the vehicle, but the applicability of their method was limited to estimating the sprung mass parameters. Yu et al. [26] identified the partial parameters of multi-axle vehicles, such as axle spacing, using wavelet analysis of global bridge responses in the bridge weigh-in-motion system. This method, like the one proposed by Rozyn et al., also could not calibrate the entire vehicle system. Bragança et al. [27] calibrated a numerical model of the freight car using track irregularities as input, experimental modal information as output, and a genetic algorithm (GA) as solver, but the method was not convenient enough because of the measurement of track irregularities. Overall, the conventional methods are costly, while these inverse methods are immature. 

Unlike the conventional methods or these immature inverse methods, the hump calibration method [28,29] is an elaborate inverse method that uses portable instruments, including smartphones and humps, for vehicle calibration. In the calibration, the first step is to record the responses with a smartphone when a vehicle passes over a hump with a known profile. In the second step, a GA [30] updates the entire vehicle parameters to match the simulated responses with the recorded measurements. It should be noted that using a designed hump instead of a road profile as input eliminates the need for road irregularities measurement. To our knowledge, a hump calibration method that relies solely on smartphones has not yet been used to estimate the parameters of multi-axle articulated vehicles, such as tractor-semitrailers. Moreover, the accuracy of the articulated vehicle model estimated by the existing method, i.e., the single-sensor hump calibration method (SHCM) [31], is questionable because tractor-semitrailers have lengths more significant than the 2-axle sedans tested in [31]. Furthermore, the quantities and positions of smartphones need to be optimized according to the articulated configuration of tractor-semitrailers. Therefore, despite the convenience and efficiency of the hump calibration method, these knowledge gaps hinder its application for the identification of articulated vehicle models.

**Figure 1:**
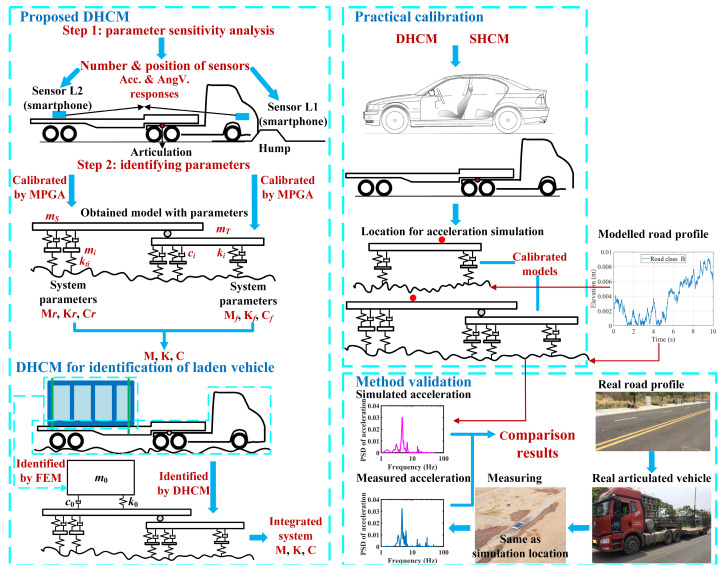
Framework of the paper.

To fill these knowledge gaps, this paper develops a novel method termed the DHCM based on the concept of the hump calibration method. The primary purpose of the proposed DHCM is to obtain an accurate model of a multi-axle articulated vehicle so that the valuable dynamic responses of the vehicle can be realistically simulated. The DHCM and its application and validation processes are illustrated in Figure 1, which includes the following task components.

- A parameter sensitivity analysis of a tractor-semitrailer model was performed to optimize the number and position of smartphone sensors. The easily accessible sensor data combined with a multiple population genetic algorithm (MPGA) made the DHCM a low-cost yet effective method for calibrating unladen articulated vehicle models. In addition, the DHCM, supplemented by analytical methods and the finite element method (FEM), was being extended for the identification of laden articulated vehicle models.
- The proposed DHCM and the existing SHCM were employed to calibrate a 2-axle sedan and a 5-axle tractor-semitrailer models. This demonstrated the superiority of the DHCM over the SHCM regarding the calibration of articulated vehicle models.
- The vertical acceleration responses were simulated by substituting the road profiles into the equations of the calibrated models. The PSDs of the simulated accelerations were then compared with those of the measured accelerations to confirm the validity of the models calibrated with the DHCM.

Overall, the results indicated that the DHCM was suitable for calibrating vehicle models with various configurations (from sedans to multi-axle articulated vehicles), while the SHCM could be used for identifying simple models such as sedan vehicles.

## 2. DHCM for Articulated Vehicle Model Identification

### 2.1. SHCM for 2-Axle Vehicle Model Identification

Despite the different types of vehicles, the equation of motion for a general pitch-plane vehicle model can be expressed as follows:

(1) $$M\overset{\cdot \;\cdot }{y}\left(t\right)+C\overset{\cdot }{y}\left(t\right)+Ky\left(t\right)=Pw\left(t\right)$$

where **M**, **C**, and **K** are the system mass, damping, and stiffness matrices; $\overset{\cdot \;\cdot }{y}\left(t\right)$, $\overset{\cdot }{y}\left(t\right)$, and **y**(*t*) are the acceleration, velocity, and displacement vectors; **P** is the force matrix; **w**(*t*) is the road displacement vector.

**Figure 2:**
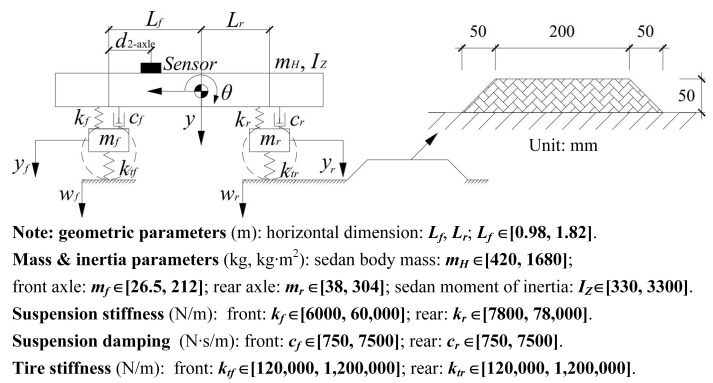
A 2-axle pitch-plane vehicle model passes over a hump.

The SHCM was mainly applied to calibrate 2-axle vehicles such as sedans and SUVs, whose motion behaviors are usually represented by a 2-axle pitch-plane vehicle model. Therefore, a 2-axle pitch-plane vehicle model (Figure 2) with four degrees of freedom (4-DOF) is an ideal example to demonstrate the principle of SHCM. The four DOFs correspond to the bouncing *y* and pitching *θ* motions of the vehicle body, and the hop motions of the two axles *y_f_* and *y_r_*. The matrices (**M**, **C**, **K**, and **P**) with the parameters for Equation (1) can be found in [31]. Since *L_f_* + *L_r_* can be measured, ten parameters (normalized by *m_H_*) are set in a vector (Equation (2)) with the subscript ‘*n*’ as follows:

(2) $$G_{v1}=\left[\begin{array}{cccccccccc} L_{f} & m_{fn} & m_{rn} & I_{Zn} & k_{fn} & k_{rn} & c_{fn} & c_{rn} & k_{tfn} & k_{trn} \end{array}\right]$$

In the measurement step of the SHCM, a 2-axle vehicle is driven over a hump with a known profile. The input—i.e., the road profile vector—is determined as **w**_2-axle_= [*w_f,hump_ w_r,hump_*]*^T^*. Meanwhile, the vertical acceleration ${\overset{\cdot \;\cdot }{y}}_{2-\mathrm{axle},\;m}$ and the pitching angular velocity ${\overset{\cdot }{\theta }}_{2-\mathrm{axle},\;m}$ of the vehicle body are measured using a smartphone (the sensor in Figure 2) located at *d*_2-axle_ from the front axle. In the simulation step of the SHCM, Equation (1) of the 2-axle vehicle is generated by randomly selecting parameters from the value ranges listed at the bottom of Figure 2. Then, the equation is solved using the Newmark method to obtain the simulated responses (vertical acceleration $\overset{\cdot \;\cdot }{y}$, pitching angular acceleration $\overset{\cdot \;\cdot }{\theta }$, and pitching angular velocity $\overset{\cdot }{\theta }$) at the center of gravity (COG) of the vehicle body. In the comparison step of the SHCM, the measured responses need to be compared with the simulated responses at the sensor location rather than those at the COG. Assuming a rigid vehicle body, the relationships between the simulated responses at the sensor location and those at the COG are described as follows:

(3) $${\overset{\cdot \;\cdot }{y}}_{2-\mathrm{axle},\;s}=\left[1\;-\left(L_{f}-d_{2-\mathrm{axle}}\right)\right]{\left[\begin{array}{cc} \overset{\cdot \;\cdot }{y} & \overset{\cdot \;\cdot }{\theta } \end{array}\right]}^{T}$$

(4) $${\overset{\cdot }{\theta }}_{2-\mathrm{axle},\;s}=\overset{\cdot }{\theta }$$

In the identification step, the deviations between the measured responses and the simulated responses at the sensor location should be minimized so that the parameters in the corresponding generated system matrix are identified as accurately as possible. The objective function representing the deviations between the measured and simulated responses is defined as follows:

(5) $$F_{D}=\sqrt{\frac{\int \left|PSD_{\mathrm{acc},\;s}\left(f\right)-PSD_{\mathrm{acc},\;m}\left(f\right)\right|df}{\int PSD_{\mathrm{acc},\;m}\left(f\right)df}}+\sqrt{\frac{\int \left|PSD_{\mathrm{angV},\;s}\left(f\right)-PSD_{\mathrm{angV},\;m}\left(f\right)\right|df}{\int PSD_{\mathrm{angV},\;m}\left(f\right)df}}$$

where *PSD*_acc,*m*_(*f*) and PSD_acc,*s*_(*f*) are the PSDs of the accelerations from the measurements and simulation, respectively; similarly, PSD_angV,*m*_(*f*) and PSD_angV,*s*_(*f*) are the measured and simulated PSDs of the angular velocities. In concrete terms, a GA is employed to identify the parameters by minimizing the objective function Equation (5). 

### 2.2. SHCM for Articulated Vehicle Model Identification

**Figure 3:**
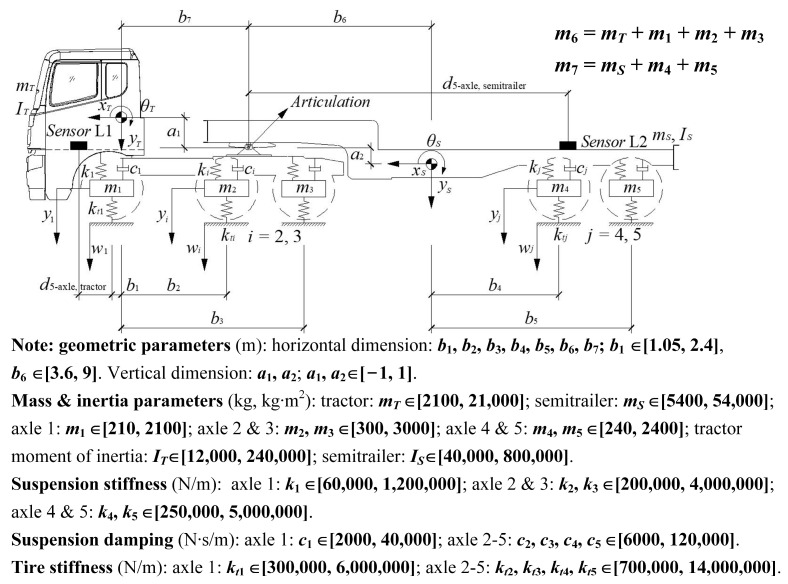
A 5-axle tractor-semitrailer vehicle model.

Regarding the modeling of a multi-axle articulated vehicle (e.g., a tractor-semitrailer) used in multidisciplinary research, a pitch-plane vehicle model with linear stiffness and damping properties was selected because its simulation results were sufficiently accurate in these studies [32,33,34]. This vehicle model has eight DOFs corresponding to the bouncing and pitching motions of the tractor body *y_T_* and *θ_T_*, the pitching motion of the semitrailer body *θ_S_*, and the hop motions of the five axles *y*_1_, *y*_2_, *y*_3_, *y*_4_, and *y*_5_, as presented in Figure 3. Note that the semitrailer of the vehicle is unladen, since in some studies on VBI dynamics [19,22] the unladen vehicles were used to excite bridge structures. The method for identifying the laden vehicle models is presented in Section 2.5. The relevant parameter ranges of the vehicle model (see Figure 3) refer to Harris [33], González [35], Yu [26], and Romero [36]. In addition, considering that a hinge was introduced to model the articulation between the tractor and semitrailer [32], the compatibility equations for the motions of the rigid tractor and semitrailer bodies are expressed as follows:

(6) $$y_{S}=y_{T}+b_{7}\theta_{T}+b_{6}\theta_{S}$$

and

(7) $$x_{S}=x_{T}+a_{1}\theta_{T}-a_{2}\theta_{S}$$

According to [34], one dynamical equation revealed an ignorable coordinate which provided the integrated equation:

(8) $$x_{T}=-m_{7}/\left(m_{6}+m_{7}\right)\times \left(a_{1}\theta_{T}-a_{2}\theta_{S}\right)$$

and

(9) $$x_{S}=m_{6}/\left(m_{6}+m_{7}\right)\times \left(a_{1}\theta_{T}-a_{2}\theta_{S}\right)$$

where *y_S_* is bouncing motion of the semitrailer body; *x_T_* and *x_S_* are fore-aft motions of the tractor and semitrailer bodies, respectively.

Similar to the equations in [32,33,34], the system mass, damping, stiffness, and force matrices **M**, **K**, **C**, and **P** in Equation (1) are derived from the equilibrium of forces and moments acting on each mass as follows:

(10) $$M=\left[\begin{array}{cccccccc} m_{T}+m_{S} & b_{7}m_{S}\; & b_{6}m_{S} & 0 & 0 & 0 & 0 & 0\\ b_{7}m_{S} & I_{T}+b_{7}{}^{2}m_{S}+\left[m_{6}m_{7}/\left(m_{6}+m_{7}\right)\right]\times a_{1}{}^{2} & b_{6}b_{7}m_{S}-\left[m_{6}m_{7}/\left(m_{6}+m_{7}\right)\right]\times a_{1}a_{2} & 0 & 0 & 0 & 0 & 0\\ b_{6}m_{S} & b_{6}b_{7}m_{S}-\left[m_{6}m_{7}/\left(m_{6}+m_{7}\right)\right]\times a_{1}a_{2} & I_{S}+b_{6}{}^{2}m_{S}+\left[m_{6}m_{7}/\left(m_{6}+m_{7}\right)\right]\times a_{2}{}^{2} & 0 & 0 & 0 & 0 & 0\\ 0 & 0 & 0 & m_{1} & 0 & 0 & 0 & 0\\ 0 & 0 & 0 & 0 & m_{2} & 0 & 0 & 0\\ 0 & 0 & 0 & 0 & 0 & m_{3} & 0 & 0\\ 0 & 0 & 0 & 0 & 0 & 0 & m_{4} & 0\\ 0 & 0 & 0 & 0 & 0 & 0 & 0 & m_{5} \end{array}\right]$$

(11) $$K=\left[\begin{array}{cccccccc} k_{1}+k_{2}+k_{3}+k_{4}+k_{5} & -b_{1}k_{1}+b_{2}k_{2}+b_{3}k_{3}+b_{7}\left(k_{4}+k_{5}\right)\; & \left(b_{6}+b_{4}\right)k_{4}+\left(b_{6}+b_{5}\right)k_{5} & -k_{1} & -k_{2} & -k_{3} & -k_{4} & -k_{5}\\ -b_{1}k_{1}+b_{2}k_{2}+b_{3}k_{3}+b_{7}\left(k_{4}+k_{5}\right) & b_{1}{}^{2}k_{1}+b_{2}{}^{2}k_{2}+b_{3}{}^{2}k_{3}+b_{7}{}^{2}\left(k_{4}+k_{5}\right) & b_{7}\left[\left(b_{6}+b_{4}\right)k_{4}+\left(b_{6}+b_{5}\right)k_{5}\right] & b_{1}k_{1} & -b_{2}k_{2} & -b_{3}k_{3} & -b_{7}k_{4} & -b_{7}k_{5}\\ \left(b_{6}+b_{4}\right)k_{4}+\left(b_{6}+b_{5}\right)k_{5} & b_{7}\left[\left(b_{6}+b_{4}\right)k_{4}+\left(b_{6}+b_{5}\right)k_{5}\right] & {\left(b_{6}+b_{4}\right)}^{2}k_{4}+{\left(b_{6}+b_{5}\right)}^{2}k_{5} & 0 & 0 & 0 & -\left(b_{6}+b_{4}\right)k_{4} & -\left(b_{6}+b_{5}\right)k_{5}\\ -k_{1} & b_{1}k_{1} & 0 & k_{1}+k_{t1} & 0 & 0 & 0 & 0\\ -k_{2} & -b_{2}k_{2} & 0 & 0 & k_{2}+k_{t2} & 0 & 0 & 0\\ -k_{3} & -b_{3}k_{3} & 0 & 0 & 0 & k_{3}+k_{t3} & 0 & 0\\ -k_{4} & -b_{7}k_{4} & -\left(b_{6}+b_{4}\right)k_{4} & 0 & 0 & 0 & k_{4}+k_{t4} & 0\\ -k_{5} & -b_{7}k_{5} & -\left(b_{6}+b_{5}\right)k_{5} & 0 & 0 & 0 & 0 & k_{5}+k_{t5} \end{array}\right]$$

(12) $$C=\left[\begin{array}{cccccccc} c_{1}+c_{2}+c_{3}+c_{4}+c_{5} & -b_{1}c_{1}+b_{2}c_{2}+b_{3}c_{3}+b_{7}\left(c_{4}+c_{5}\right)\; & \left(b_{6}+b_{4}\right)c_{4}+\left(b_{6}+b_{5}\right)c_{5} & -c_{1} & -c_{2} & -c_{3} & -c_{4} & -c_{5}\\ -b_{1}c_{1}+b_{2}c_{2}+b_{3}c_{3}+b_{7}\left(c_{4}+c_{5}\right) & b_{1}{}^{2}c_{1}+b_{2}{}^{2}c_{2}+b_{3}{}^{2}c_{3}+b_{7}{}^{2}\left(c_{4}+c_{5}\right) & b_{7}\left[\left(b_{6}+b_{4}\right)c_{4}+\left(b_{6}+b_{5}\right)c_{5}\right] & b_{1}c_{1} & -b_{2}c_{2} & -b_{3}c_{3} & -b_{7}c_{4} & -b_{7}c_{5}\\ \left(b_{6}+b_{4}\right)c_{4}+\left(b_{6}+b_{5}\right)c_{5} & b_{7}\left[\left(b_{6}+b_{4}\right)c_{4}+\left(b_{6}+b_{5}\right)c_{5}\right] & {\left(b_{6}+b_{4}\right)}^{2}c_{4}+{\left(b_{6}+b_{5}\right)}^{2}c_{5} & 0 & 0 & 0 & -\left(b_{6}+b_{4}\right)c_{4} & -\left(b_{6}+b_{5}\right)c_{5}\\ -c_{1} & b_{1}c_{1} & 0 & c_{1} & 0 & 0 & 0 & 0\\ -c_{2} & -b_{2}c_{2} & 0 & 0 & c_{2} & 0 & 0 & 0\\ -c_{3} & -b_{3}c_{3} & 0 & 0 & 0 & c_{3} & 0 & 0\\ -c_{4} & -b_{7}c_{4} & -\left(b_{6}+b_{4}\right)c_{4} & 0 & 0 & 0 & c_{4} & 0\\ -c_{5} & -b_{7}c_{5} & -\left(b_{6}+b_{5}\right)c_{5} & 0 & 0 & 0 & 0 & c_{5} \end{array}\right]$$

(13) $$P={\left[\begin{array}{cccccccc} 0 & 0 & 0 & k_{t1} & 0 & 0 & 0 & 0\\ 0 & 0 & 0 & 0 & k_{t2} & 0 & 0 & 0\\ 0 & 0 & 0 & 0 & 0 & k_{t3} & 0 & 0\\ 0 & 0 & 0 & 0 & 0 & 0 & k_{t4} & 0\\ 0 & 0 & 0 & 0 & 0 & 0 & 0 & k_{t5} \end{array}\right]}^{T}$$

The parameters (normalized by the tractor body mass *m_T_*) of the tractor-semitrailer model are set in vector form as follows:

(14) $$G_{v2}=[m_{Sn}\;m_{1n}\;m_{2n}\;m_{3n}\;m_{4n}\;m_{5n}\;I_{Tn}\;I_{Sn}\;k_{1n}\;k_{2n}\;k_{3n}\;k_{4n}\;k_{5n}\;c_{1n}\;c_{2n}\;c_{3n}\;c_{4n}\;c_{5n}\;k_{t1n}\;k_{t2n}\;k_{t3n}\;k_{t4n}\;k_{t5n}\;a_{1}\;a_{2}\;b_{1}\;b_{6}]$$

**Figure 4:**
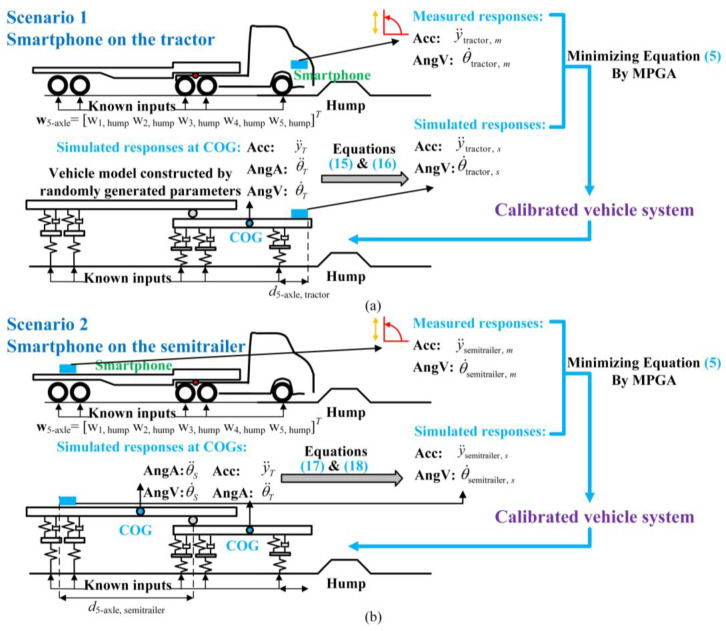
The SHCM for the tractor-semitrailer model. (**a**) Scenario 1; (**b**) Scenario 2.

Unlike the calibration of 2-axle vehicles, it is necessary to determine whether a smartphone will be installed on the tractor or on the semitrailer before using the SHCM to identify a 5-axle tractor-semitrailer model. As shown in Figure 4a, the relationships between the simulated responses at the sensor location and the responses at the COG of the tractor are described by Equations (15) and (16) when a smartphone is installed on the tractor:

(15) $${\overset{\cdot \;\cdot }{y}}_{\mathrm{tractor},\;s}=\left[\begin{array}{cc} 1 & -\left(b_{1}-d_{5-\mathrm{axle},\;\mathrm{tractor}}\right) \end{array}\right]{\left[\begin{array}{cc} {\overset{\cdot \;\cdot }{y}}_{T} & {\overset{\cdot \;\cdot }{\theta }}_{T} \end{array}\right]}^{T}$$

(16) $${\overset{\cdot }{\theta }}_{\mathrm{tractor},\;s}={\overset{\cdot }{\theta }}_{T}$$

Otherwise, as shown in Figure 4b, based on Equation (6), the simulated responses at the sensor location on the semitrailer are related to the responses at the COGs of the tractor and semitrailer through:

(17) $${\overset{\cdot \;\cdot }{y}}_{\mathrm{semitrailer},\;s}=\left[\begin{array}{ccc} 1 & b_{7} & d_{5-\mathrm{axle},\;\mathrm{semitrailer}} \end{array}\right]{\left[\begin{array}{cc} {\overset{\cdot \;\cdot }{y}}_{T} & \begin{array}{cc} {\overset{\cdot \;\cdot }{\theta }}_{T} & {\overset{\cdot \;\cdot }{\theta }}_{S} \end{array} \end{array}\right]}^{T}\;$$

(18) $${\overset{\cdot }{\theta }}_{\mathrm{semitrailer},\;s}={\overset{\cdot }{\theta }}_{S}$$

The parameter vector **G***_v_*_2_ is obtained by minimizing the objective function. Due to the different expressions of Equations (15) and (17), there are significant discrepancies in the parameters of the calibrated models using the responses on the tractor and the semitrailer, respectively. Essentially, the different correlations of one parameter with the responses at the two locations lead to significant discrepancies between the two sets of parameters. Therefore, a parameter sensitivity analysis is performed to quantify the correlations between the parameters and the responses so that we can find out which parameters of a tractor-semitrailer model can be accurately determined based on the responses of a smartphone installed at which location of the tractor-semitrailer.

### 2.3. Parameter Sensitivity Analysis

Assuming that all parameters of a vehicle model are determined, the responses of the vehicle model can be calculated when the vehicle is driven over the designed hump. The sensitivities of the responses of the vehicle model to a perturbation in a parameter provide a quantification of the contribution of the parameter to the responses [29,37]. Suppose a parameter, *α*, related only to stiffness (K), is perturbed by Δ*α*. In that case, the perturbed equation, i.e., Equation (19), is obtained by differentiating both sides of Equation (1) with respect to *α* as follows:

(19) $$M\left\{\partial \overset{\cdot \;\cdot }{y}\left(t\right)/\partial \alpha \right\}+C\left\{\partial \overset{\cdot }{y}\left(t\right)/\partial \alpha \right\}+K\left\{\partial y\left(t\right)/\partial \alpha \right\}=\left\{\partial P/\partial \alpha \right\}w\left(t\right)-\left\{\partial K/\partial \alpha \right\}y\left(t\right)$$

where $\left\{\partial \overset{\cdot \;\cdot }{y}\left(t\right)/\partial \alpha \right\}$, $\left\{\partial \overset{\cdot }{y}\left(t\right)/\partial \alpha \right\}$, and $\left\{\partial y\left(t\right)/\partial \alpha \right\}$ are the sensitivities of acceleration, velocity, and displacement responses to *α*. As acceleration sensitivity is the most commonly used sensitivity, and the sensitivity described below refers only to acceleration sensitivity.

In short, the sensitivity of a response to a parameter quantifies the correlation between the parameter and the vehicle response. The high sensitivity of a parameter indicates that the parameter significantly affects the response of the vehicle model, which in turn implies that a parameter that possesses a strong correlation with the response can be accurately identified from the response. Specifically, two ways exist to install the sensors in the SHCM to calibrate a 5-axle tractor-semitrailer model. A parameter sensitivity analysis explores which parameters can be more accurately estimated using the vertical acceleration of a sensor installed on the tractor or semitrailer.

**Figure 5:**
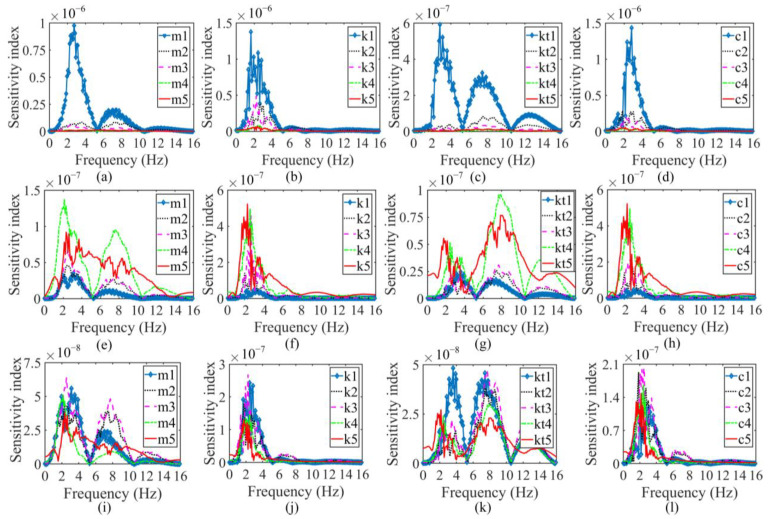
Sensitivity indices. (**a**) Mass at L1; (**b**) suspension stiffness at L1; (**c**) tire stiffness at L1; (**d**) damping at L1; (**e**) mass at L2; (**f**) suspension stiffness at L2; (**g**) tire stiffness at L2; (**h**) damping at L2; (**i**) mass at L2′; (**j**) suspension stiffness at L2′; (**k**) tire stiffness at L2′; (**l**) damping at L2′.

Based on the above discussion, a set of parameters for a tractor-semitrailer model sourced from [26,33] were normalized by *m_T_* and provided as: [*b*_1_, *b*_2_, *b*_3_, *b*_4_, *b*_5_, *b*_6_, *b*_7_, *a*_1_, *a*_2_] = [1.7, 1.5, 2.5, 2.4, 3.5, 5.5, 1.8, 0.2, −0.5], [*m_T_*, *m_S_*, *I_T_*, *I_S_*, *m*_1_, *m*_2_, *m*_3_, *m*_4_, *m*_5_] = [1, 4.5, 10, 40, 0.175, 0.25, 0.25, 0.3, 0.3], [*k*_1_, *k*_2_, *k*_3_, *k*_4_, *k*_5_] = [100, 150, 150, 187.5, 187.5], [c_1_, c_2_, c_3_, c_4_, c_5_] = [12.5, 12.5, 12.5, 12.5, 12.5], and [*kt*_1_, *kt*_2_, *kt*_3_, *kt*_4_, *kt*_5_] = [437.5, 875, 875, 875, 875]. As for the basis for selecting the position L1 of the sensor on the tractor and the position L2 of the sensor on the semitrailer, the position for L1 is determined at *d*_5-axis,tractor_ = 0 m from axle 1, since the space for installing the sensor L1 on the floor of the tractor is rather limited. A comparison of Figure 5i–l with Figure 5e–h also shows that if the sensor is installed on the semitrailer at a position closer to the articulation point, such as the position L2′ at *d*_5-axle,semitrailer′_ = 2.5 m from the articulation point, the sensitivities of the sensor response at L2′ for the parameters from axle 1 to axle 5 remain approximately at the same level and the magnitude is lower than that of the sensitivities of the sensor response at L2 (at *d*_5-axle,semitrailer_ = 8.45 m from the articulation and between axle 4 and 5) for the parameters from axle 2 to axle 5. The further away the sensor L2 is from the articulation, the more accurate the estimate of the parameters from axle 2 to axle 5—the position L2 is determined accordingly. The sensitivities of the vertical accelerations at L1 and L2 to the mass, damping, and stiffness parameters of the vehicle model are calculated using Equations (15)–(19). The relevant sensitivity indices are expressed in the frequency domain as follows:

(20) $$Sensitivity\;index=\sqrt{PSD_{\mathrm{sensitivity}}\left(f\right)}$$

As can be seen in Figure 5a–d, the sensitivities of the response of the sensor at L1 indicate that the parameters, including mass, suspension stiffness, tire stiffness, and damping parameters, from axle 1 have a strong influence on the vertical acceleration, while the parameters from axle 2 to axle 5 correlate only weakly with the vertical acceleration. In contrast, the sensitivities of the sensor response at L2, shown in Figure 5e–h, reveal that the vertical acceleration at L2 is significantly affected by the parameters from axle 2 to axle 5, but not by the parameters from axle 1. Therefore, regardless of the position of the sensors, the SHCM cannot accurately determine the complete set of parameters for tractor-semitrailer models. To calibrate a tractor-semitrailer model accurately, at least one sensor should be installed separately at L1 and L2. 

### 2.4. The Proposed DHCM

The results of the parameter sensitivity analysis shed light on the proposed DHCM, which is able to more accurately calibrate the model of a multi-axle articulated vehicle using the minimum number of two sensors installed on the tractor and semitrailer, respectively. For a 5-axle vehicle model, the optimal sensor positions are (1) above axle 1 for Sensor 1 and (2) between axle 4 and axle 5 for Sensor 2. 

**Figure 6:**
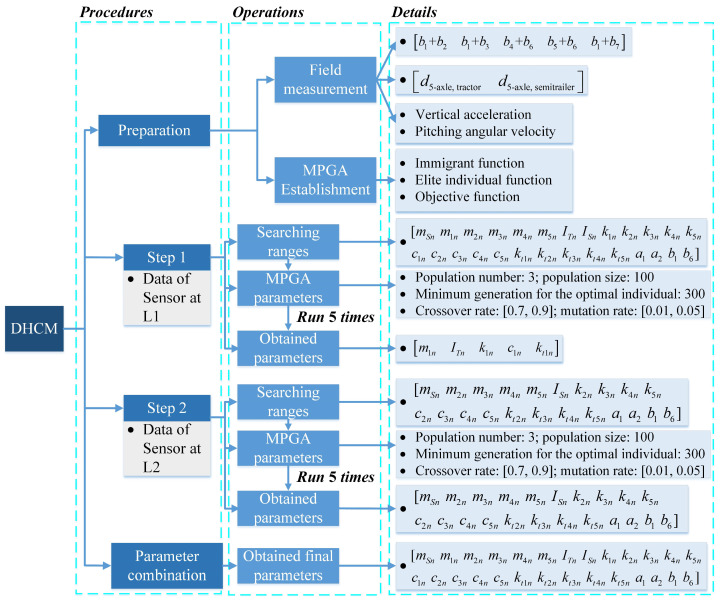
The DHCM implementation.

The procedure for the DHCM implementation is presented in Figure 6. In the preparation phase, geometric parameters such as the wheelbases, the position of the articulation, and the position of the sensors are measured. Due to the large number of parameters to be identified, an MPGA [38] is proposed to replace the GA and overcome the problem of premature convergence in the following minimization process. Figure 6 also shows the configuration of MPGA. The process of MPGA involves selection, crossover, mutation, and migration. In selection, the population number is 3, the population size is 100, the generation is 300, and each chromosome *i* (*i* = 1, 2, …, 100) is a vector of vehicle parameters. The selection is based on the fitness values (*F_i_*, the smaller the better) of each chromosome *i* to select the chromosome that represents the better possible solutions. *F_i_* is therefore the most important function in MAGA and is defined as

(21) $$F_{i}=2\left(Pos_{i}-1\right)/99$$

where *Pos_i_* is derived from the objective function value (*F_Di_*, according to Equation (5)) of chromosome *i*. If the vector **F***_D_* = [*F_D_*_1_ *F_D_*_2_ … *F_Di_* … *F_D_*_100_] is positively ordered as vector **Pos**, then *Pos_i_* is the position number of *F_Di_* in **Pos**. In crossover (crossover rate within [0.7, 0.9]), the parents are crossed to form a new generation. In mutation (mutation rate within [0.01, 0.05]), one or more values of a gene on a chromosome are changed to maintain genetic diversity in the next generation. During migration, the new generation replaces the old subpopulations using the immigration function [39]. Note that the elite individuals are selected in this step using the elite individual function. After the migration, and if the termination condition is met, the MPGA stops and returns the best solution of the populations. Otherwise, the algorithm continues until the 300th generation is reached.

Returning to the first step of the DHCM, the vector **G***_v_*_2_, as shown in Equation (14), is calibrated using the data from Sensor 1. Then the results of the parameters from axle 1 are entered into a vector as **G***_v_*_2,axle1_ = [*m*_1*n*_ *I_Tn_ k*_1*n*_ *c*_1*n*_ *k_t_*_1*n*_]. In the second step, the parameters in **G***_v_*_2,axle1_ are known, and the remaining parameters are calibrated using the data from Sensor 2 and entered into a vector as **G***_v_*_2,axle2-5_ as follows:

(22) $$G_{v2,\;\mathrm{axle}2-5}=[m_{Sn}\;m_{2n}\;m_{3n}\;m_{4n}\;m_{5n}\;I_{Sn}\;k_{2n}\;k_{3n}\;k_{4n}\;k_{5n}\;c_{2n}\;c_{3n}\;c_{4n}\;c_{5n}\;k_{t2n}\;k_{t3n}\;k_{t4n}\;k_{t5n}\;a_{1}\;a_{2}\;b_{1}\;b_{6}]$$

In the parameter combination step, **G***_v_*_2,axle1_ and **G***_v_*_2,axle2-5_ are integrated to obtain **G***_v_*_2_.

### 2.5. DHCM for Laden Vehicle Model Identification

Articulated vehicles are designed to transport objects (loads) that strongly influence the mass and inertia properties of the semitrailer as well as the position of the semitrailer’s COG. From a conventional point of view, the calibration of laden articulated vehicles requires laborious procedures that do not appear practicable due to the variety of objects transported. However, the system matrices **M**, **K**, **C**, and **P** for the laden articulated vehicles can be determined on the basis of the unladen articulated vehicles identified with the DHCM and supplemented by analytical methods or the FEM. Concretely, the identification of laden articulated vehicles is divided into two scenarios, the first for the transportation of neatly stacked objects such as food (where the transported objects only have mass properties) and the second for the transportation of individual objects such as building modules (where the transported objects have mass, stiffness and damping properties). Regardless of the scenarios, the prerequisite for identifying a laden vehicle model is that the parameters of the same unladen vehicle have been identified with the DHCM, i.e., Equation (14) for the tractor-semitrailer model in Figure 3 is known.

**Figure 7:**
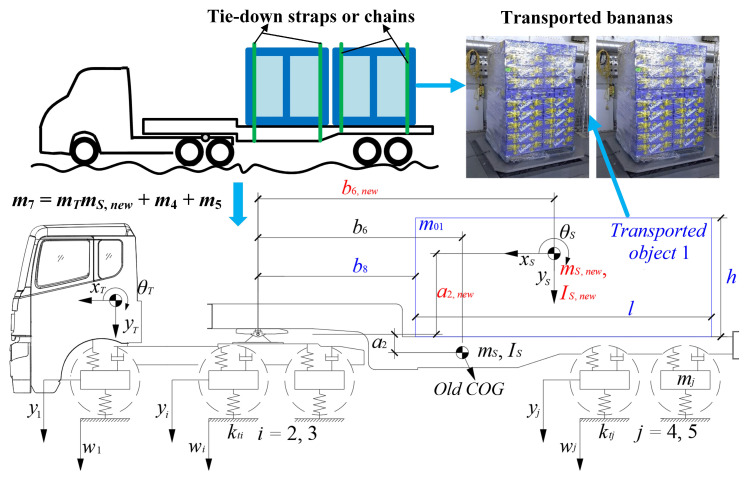
Laden vehicle model identification in the first scenario (using bananas in [6] as an example).

In the first scenario, shown in Figure 7, it is assumed that the transported object is a rectangle with measurable parameters including a horizontal distance *b*_8_ from the articulation point, a length *l*, a height *h*, and a mass *m*_01_. Then the transported object and the semitrailer are merged into one unit with new parameters including the mass *m*_S,new_, the pitch moment of inertia *I*_S,new_, the horizontal distance of the COG *b*_6,new_ from the articulation point, and the vertical distance of the COG *a*_2,new_ from the articulation point, which are determined as follows:

(23) $$\left\{\begin{array}{l} m_{S,new}=m_{Sn}+m_{01}/m_{T}\\ I_{S,new}=I_{Sn}+m_{01}(l^{2}+h^{2})/(12m_{T})\\ b_{6,new}=((b_{8}+l/2)m_{01}/m_{T}+b_{6}m_{Sn})/(m_{01}/m_{T}+m_{Sn})\\ a_{2,new}=((h/2-a_{2})m_{01}/m_{T}+a_{2}m_{Sn})/(m_{01}/m_{T}+m_{Sn}) \end{array}\right.$$

where *m*_Sn_, *I*_Sn_, *b*_6_, and *a*_2_ are known parameters from Equation (14); *m_T_* is a known parameter for the mass of the tractor. Finally, *m*_Sn_, *I*_Sn_, *b*_6_, and *a*_2_ in Equation (14) are replaced by *m*_S,new_, *I*_S,new_, *b*_6,new_, and *a*_2,new_ correspondingly to form the matrices **M**, **K**, **C** and **P** for the laden tractor-semitrailer model according to Equations (10)–(13).

**Figure 8:**
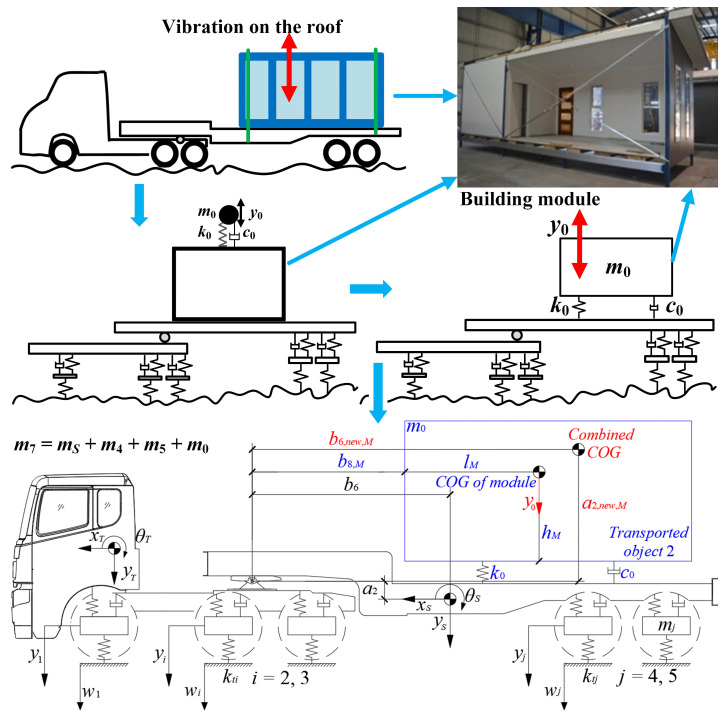
Laden vehicle model identification in the second scenario (using the building module in [12] as an example).

In the second scenario, shown in Figure 8, the cargo used as an example is a previously introduced building module [9]. During transportation, vertical vibrations *y*_0_ occur on the roof of the building module, which represent an additional DOF for the entire system. This single-DOF transported object (with the unknown parameters of mass *m*_0_, stiffness *k*_0_, and damping *c*_0_) shifts the horizontal distance *b*_6,new,*M*_ and the vertical distance *a*_2, new,*M*_ of the combined COG of the semitrailer and the building module from the articulation point. The unidentified parameters are therefore *m*_0_, *k*_0_, *c*_0_, *b*_6,new,*M*_, and *a*_2,new,*M*_.

Since the transported object varies from a building module to any load of similar size, it is time-consuming to determine the parameters *m*_0_, *k*_0_, and *c*_0_ via experimental approaches. A cost-saving alternative to experimental approaches is the FEM, which can also be illustrated using the example of a building module. Sharafi et al. [40] have demonstrated that the FE model built with ABAQUS software accurately simulates the horizontal dynamic properties of a building module compared with the experimental results. Here, we used ABAQUS to determine *m*_0_, *k*_0_, *c*_0_, and COG of the building module in [12] (where its configurations and material properties were elaborated). In addition, the four corners of the FE model were constrained along the three translation directions according to [12]. 

**Figure 9:**
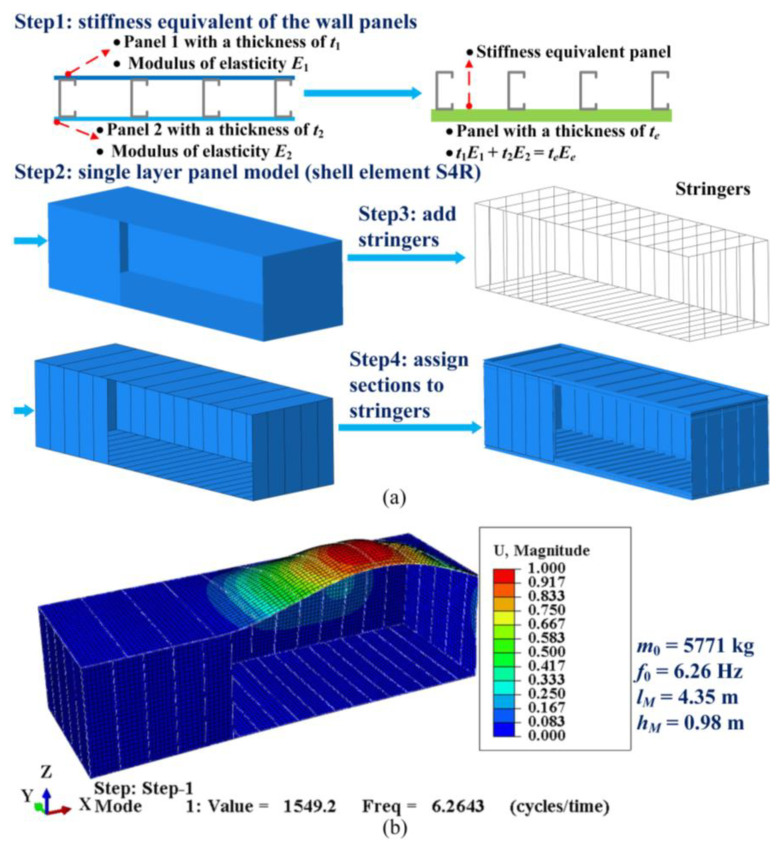
Modeling process and simulation results of the FEM. (**a**) The FE model; (**b**) the dynamic properties of the building module.

Due to the limited space, we briefly describe the modeling process and the simulation results of the FEM (see Figure 9). As can be seen in Figure 9a, based on the vertical stiffness equivalence, the different wall panels corresponded to a single-layer panel with a thickness of (*t*_1_*E*_1_ + *t*_2_*E*_2_)/*E_e_*. The sheathing of the building module was then modeled with the S4R shell element. Finally, the steel frame elements on the sheathing were created using stringers with cross-sectional profiles. As shown in Figure 9b, a linear perturbation analysis was performed with ABAQUS to determine the position of the COG, the total mass *m*_0_, the principal vertical vibration mode, and the corresponding fundamental frequency *f*_0_ of the building module. Accordingly, the mass parameter *m*_0_ = 5771 kg, the stiffness parameter *k*_0_ = (2π*f*_0_)^2^*m*_0_ = 8931 kN/m, the damping parameter *c*_0_ = 9.1 kN s/m (Rayleigh damping [**C**] = α[**M**] + β[**K**]), the horizontal parameter of the COG *l_M_* = 4.35 m, and the vertical parameter of the COG *h_M_* = 0.98 m. According to Figure 8, the parameters *b*_6,new,*M*_ and *a*_2,new,*M*_ are expressed as follows:

(24) $$\left\{\begin{array}{l} b_{6,new,M}=(b_{9}m_{0}/m_{T}+b_{6}m_{Sn})/(m_{0}/m_{T}+m_{Sn})\\ a_{2,new,M}=((h_{M}-a_{2})m_{0}/m_{T}+a_{2}m_{Sn})/(m_{01}/m_{T}+m_{Sn}) \end{array}\right.$$

where *b*_9_ = *l_M_* + *b*_8,*M*_; where the horizontal distance *b*_8,*M*_ of the building module from the articulation point is measurable.

As already mentioned, the additional parameters for the laden articulated vehicle model are determined. These parameters are then used together with the parameters in Equation (14) to construct the equation of motion (Equation (1)) of the laden articulated vehicle model. According to Figure 8, the displacement vector **y**(*t*) in Equation (1) is [*y_t_ θ_T_ θ_S_ y*_1_ *y*_2_ *y*_3_ *y*_4_ *y*_5_ *y*_0_]*^T^*. Based on the new equilibrium of forces and moments acting on each mass, the system matrices **M**, **K**, **C**, and **P** for the laden tractor-semitrailer model are rewritten as follows:

(25) $$M=\left[\begin{array}{ccccccccc} \left(m_{T}+m_{S}\right) & b_{7}m_{S} & b_{6}m_{S} & 0 & 0 & 0 & 0 & 0 & 0\\ b_{7}m_{S} & \left(I_{T}+b_{7}{}^{2}m_{S}+\left(m_{6}m_{7}/\left(m_{6}+m_{7}\right)\right)\times a_{1}{}^{2}\right) & \left(b_{6}b_{7}m_{S}-\left(m_{6}m_{7}/\left(m_{6}+m_{7}\right)\right)\times a_{1}a_{2}\right) & 0 & 0 & 0 & 0 & 0 & 0\\ b_{6}m_{S} & \left(b_{6}b_{7}m_{S}-\left(m_{6}m_{7}/\left(m_{6}+m_{7}\right)\right)\times a_{1}a_{2}\right) & \left(I_{S}+b_{6}{}^{2}m_{S}+\left(m_{6}m_{7}/\left(m_{6}+m_{7}\right)\right)\times a_{2}{}^{2}\right) & 0 & 0 & 0 & 0 & 0 & 0\\ 0 & 0 & 0 & m_{1} & 0 & 0 & 0 & 0 & 0\\ 0 & 0 & 0 & 0 & m_{2} & 0 & 0 & 0 & 0\\ 0 & 0 & 0 & 0 & 0 & m_{3} & 0 & 0 & 0\\ 0 & 0 & 0 & 0 & 0 & 0 & m_{4} & 0 & 0\\ 0 & 0 & 0 & 0 & 0 & 0 & 0 & m_{5} & 0\\ 0 & 0 & 0 & 0 & 0 & 0 & 0 & 0 & m_{0} \end{array}\right]$$

(26) $$K=\left[\begin{array}{ccccccccc} k_{1}+k_{2}+k_{3}+k_{4}+k_{5}+k_{0} & -b_{1}k_{1}+b_{2}k_{2}+b_{3}k_{3}+b_{7}\left(k_{4}+k_{5}+k_{0}\right) & \left(b_{6}+b_{4}\right)k_{4}+\left(b_{6}+b_{5}\right)k_{5}+b_{9}k_{0} & -k_{1} & -k_{2} & -k_{3} & -k_{4} & -k_{5} & -k_{0}\\ -b_{1}k_{1}+b_{2}k_{2}+b_{3}k_{3}+b_{7}\left(k_{4}+k_{5}+k_{0}\right) & b_{1}{}^{2}k_{1}+b_{2}{}^{2}k_{2}+b_{3}{}^{2}k_{3}+b_{7}{}^{2}\left(k_{4}+k_{5}+k_{0}\right) & b_{7}\left(\left(b_{6}+b_{4}\right)k_{4}+\left(b_{6}+b_{5}\right)k_{5}+b_{9}k_{0}\right) & b_{1}k_{1} & -b_{2}k_{2} & -b_{3}k_{3} & -b_{7}k_{4} & -b_{7}k_{5} & -b_{7}k_{0}\\ \left(b_{6}+b_{4}\right)k_{4}+\left(b_{6}+b_{5}\right)k_{5}+b_{9}k_{0} & b_{7}\left(\left(b_{6}+b_{4}\right)k_{4}+\left(b_{6}+b_{5}\right)k_{5}+b_{9}k_{0}\right) & {\left(b_{6}+b_{4}\right)}^{2}k_{4}+{\left(b_{6}+b_{5}\right)}^{2}k_{5}+b_{9}{}^{2}k_{0} & 0 & 0 & 0 & -\left(b_{6}+b_{4}\right)k_{4} & -\left(b_{6}+b_{5}\right)k_{5} & -b_{9}k_{0}\\ -k_{1} & b_{1}k_{1} & 0 & k_{1}+k_{t1} & 0 & 0 & 0 & 0 & 0\\ -k_{2} & -b_{2}k_{2} & 0 & 0 & k_{2}+k_{t2} & 0 & 0 & 0 & 0\\ -k_{3} & -b_{3}k_{3} & 0 & 0 & 0 & k_{3}+k_{t3} & 0 & 0 & 0\\ -k_{4} & -b_{7}k_{4} & -\left(b_{6}+b_{4}\right)k_{4} & 0 & 0 & 0 & k_{4}+k_{t4} & 0 & 0\\ -k_{5} & -b_{7}k_{5} & -\left(b_{6}+b_{5}\right)k_{5} & 0 & 0 & 0 & 0 & k_{5}+k_{t5} & 0\\ -k_{0} & -b_{7}k_{0} & -b_{9}k_{0} & 0 & 0 & 0 & 0 & 0 & k_{0} \end{array}\right]$$

(27) $$C=\left[\begin{array}{ccccccccc} c_{1}+c_{2}+c_{3}+c_{4}+c_{5}+c_{0} & -b_{1}c_{1}+b_{2}c_{2}+b_{3}c_{3}+b_{7}\left(c_{4}+c_{5}+c_{0}\right) & \left(b_{6}+b_{4}\right)c_{4}+\left(b_{6}+b_{5}\right)c_{5}+b_{9}c_{0} & -c_{1} & -c_{2} & -c_{3} & -c_{4} & -c_{5} & -c_{0}\\ -b_{1}c_{1}+b_{2}c_{2}+b_{3}c_{3}+b_{7}\left(c_{4}+c_{5}+c_{0}\right) & b_{1}{}^{2}c_{1}+b_{2}{}^{2}c_{2}+b_{3}{}^{2}c_{3}+b_{7}{}^{2}\left(c_{4}+c_{5}+c_{0}\right) & b_{7}\left(\left(b_{6}+b_{4}\right)c_{4}+\left(b_{6}+b_{5}\right)c_{5}+b_{9}c_{0}\right) & b_{1}c_{1} & -b_{2}c_{2} & -b_{3}c_{3} & -b_{7}c_{4} & -b_{7}c_{5} & -b_{7}c_{0}\\ \left(b_{6}+b_{4}\right)c_{4}+\left(b_{6}+b_{5}\right)c_{5}+b_{9}c_{0} & b_{7}\left(\left(b_{6}+b_{4}\right)c_{4}+\left(b_{6}+b_{5}\right)c_{5}+b_{9}c_{0}\right) & {\left(b_{6}+b_{4}\right)}^{2}c_{4}+{\left(b_{6}+b_{5}\right)}^{2}c_{5}+b_{9}{}^{2}c_{0} & 0 & 0 & 0 & -\left(b_{6}+b_{4}\right)c_{4} & -\left(b_{6}+b_{5}\right)c_{5} & -b_{9}c_{0}\\ -c_{1} & b_{1}c_{1} & 0 & c_{1} & 0 & 0 & 0 & 0 & 0\\ -c_{2} & -b_{2}c_{2} & 0 & 0 & c_{2} & 0 & 0 & 0 & 0\\ -c_{3} & -b_{3}c_{3} & 0 & 0 & 0 & c_{3} & 0 & 0 & 0\\ -c_{4} & -b_{7}c_{4} & -\left(b_{6}+b_{4}\right)c_{4} & 0 & 0 & 0 & c_{4} & 0 & 0\\ -c_{5} & -b_{7}c_{5} & -\left(b_{6}+b_{5}\right)c_{5} & 0 & 0 & 0 & 0 & c_{5} & 0\\ -c_{0} & -b_{7}c_{0} & -b_{9}c_{0} & 0 & 0 & 0 & 0 & 0 & c_{0} \end{array}\right]$$

(28) $$P=\left[\begin{array}{ccccc} 0 & 0 & 0 & 0 & 0\\ 0 & 0 & 0 & 0 & 0\\ 0 & 0 & 0 & 0 & 0\\ k_{t1} & 0 & 0 & 0 & 0\\ 0 & k_{t2} & 0 & 0 & 0\\ 0 & 0 & k_{t3} & 0 & 0\\ 0 & 0 & 0 & k_{t4} & 0\\ 0 & 0 & 0 & 0 & k_{t5}\\ 0 & 0 & 0 & 0 & 0 \end{array}\right]$$

Overall, the unladen articulated vehicle models calibrated with the DHCM form the basis for identifying the laden articulated vehicle models. Considering that all unknown parameters of a laden articulated vehicle model are identified, the dynamic responses of both the vehicle and the transported object can be simulated according to Equations (1) and (25)–(28).

## 3. Calibration Results

The idea behind our research is that given the fact that the same unladen tractor-semitrailer is used to transport a variety of objects (from a building module to a bulldozer), only the parameters of the unladen vehicle need to be calibrated using the DHCM via a real experiment and the parameters of the objects transported on it are determined using a cost-saving FEM. Due to the importance of the unladen vehicle model, the proposed DHCM and the existing SHCM were used for the practical calibration of an unladen 2-axle sedan model and an unladen 5-axle tractor-semitrailer model. It should be noted that the DHCM can be used to calibrate a 2-axle vehicle model by placing the two sensors near the front and rear axles, respectively. Furthermore, the calibration of a laden vehicle carrying a specific load is not representative given the variety of objects transported, although the vehicle itself remains unchanged.

### 3.1. Instrumentation

**Figure 10:**
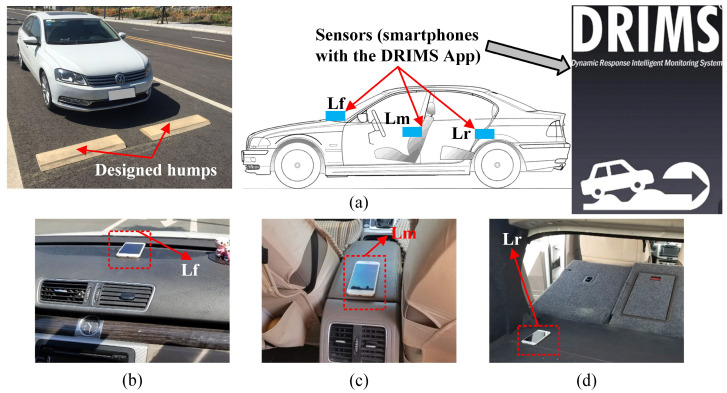
Sensor layout for sedan vehicle model calibration. (**a**) Sensor layout; (**b**) position of Lm; (**c**) position of Lf; (**d**) position of Lr.

The measurement device was an Apple iPhone with an iOS application called DRIMS (Figure 10a). The smartphone application can simultaneously record acceleration and angular velocity at a sampling frequency of 100 Hz and a GPS signal at a frequency of 1 Hz. Compared with the acceleration and angular velocity signals recorded with a dedicated accelerometer and gyroscope, the signals acquired with the smartphone exhibited good consistencies in the frequency range of 0.15–40 Hz [31]. Considering that the natural frequencies corresponding to the bouncing, pitching, and axle hop motion modes of most articulated vehicles are below 25 Hz, smartphones are suitable wireless sensors for capturing the dynamic responses of vehicles.

### 3.2. Calibration of the 2-Axle Vehicle Model

As an idealized form to simplify most vehicle types, a 2-axle vehicle model was calibrated using the SHCM and the DHCM to illustrate the differences between the two calibration methods. The calibrated 2-axle vehicle models were also employed to discuss the application scopes of the two calibration methods in Section 4. 

The test vehicle was an ordinary sedan, as shown in Figure 10a, driven at a constant speed of 2.5 m/s during the test. It also shows that two identical humps with the intended dimensions (see Figure 2) were placed on a road with a relatively smooth surface. Note that a higher driving speed was avoided, as a fast suspension movement can lead to significant nonlinearity in the vehicle responses and bouncing of the vehicle wheel off the hump, which are not considered in the model. Thus, the vehicle speeds in the calibrations remained below 10 km/h (2.8 m/s) [31]. As detailed in Figure 10a–d), Sensor Lm, located near the middle of the vehicle, was used for the SHCM, while Sensors Lf and Lr, located near the front and rear of the vehicle, were used for the DHCM. Note that the data from the Sensor Lf and Sensor Lr were also available for the SHCM, so the effect of the different sensor positions on the accuracy of the vehicle model calibrated with the SHCM was considered. In addition, the measurements of the preparation phase provided us with information about the wheelbase, 2.8 m, and the positions of Sensor Lm, Sensor Lf, and Sensor Lr (*d*_2-axle_ = 1.34, 0.53, and 2.61 m, respectively).

**Figure 11:**
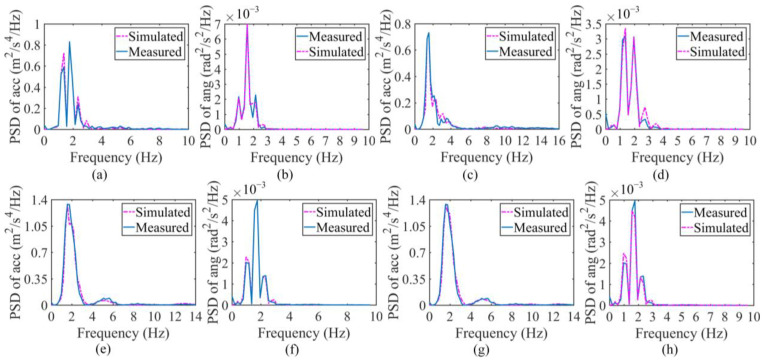
Comparisons for 2-axle vehicle calibration. (**a**) Accelerations at Lm for the SHCM; (**b**) angular velocities at Lm for the SHCM; (**c**) accelerations at Lf for the SHCM; (**d**) angular velocities at Lf for the SHCM; (**e**) accelerations at Lr for the SHCM; (**f**) angular velocities at Lr for the SHCM; (**g**) accelerations for the DHCM; (**h**) angular velocities for the DHCM. Note: (**g**,**h**) only illustrate the comparisons in step 2 of the DHCM, since the comparisons in step 1 of the DHCM are the same as (**c**,**d**).

The SHCM and DHCM were implemented by minimizing the objective function, i.e., Equation (5), using GA and MPGA, respectively. The excellent agreement between the PSD curves of the simulated responses and the PSD curves of the measured responses indicates that the minimization of the objective function is achieved in the last step of the SHCM and DHCM so that the vehicle parameters can be identified. Therefore, the comparisons of PSDs between the simulated and measured responses for the SHCM and DHCM are presented in Figure 11a–h, respectively. Despite the different sensor positions, the PSDs of the simulated and measured vehicle responses exhibit good consistencies. Accordingly, the normalized parameters can be obtained from the SHCM and DHCM to further generate the 2-axle vehicle systems, i.e., four calibrated vehicle models labeled SHCM-M, SHCM-F, SHCM-R, and DHCM-F&R. The normalized parameters of the identified vehicle models are listed in Table A1. The different dynamic characteristics between the models calibrated with SHCM (SHCM-M, SHCM-F, and SHCM-R) and the model calibrated with the DHCM (DHCM-F&R) were discussed in Section 3.4. 

### 3.3. Calibration of the Articulated Vehicle Model

**Figure 12:**
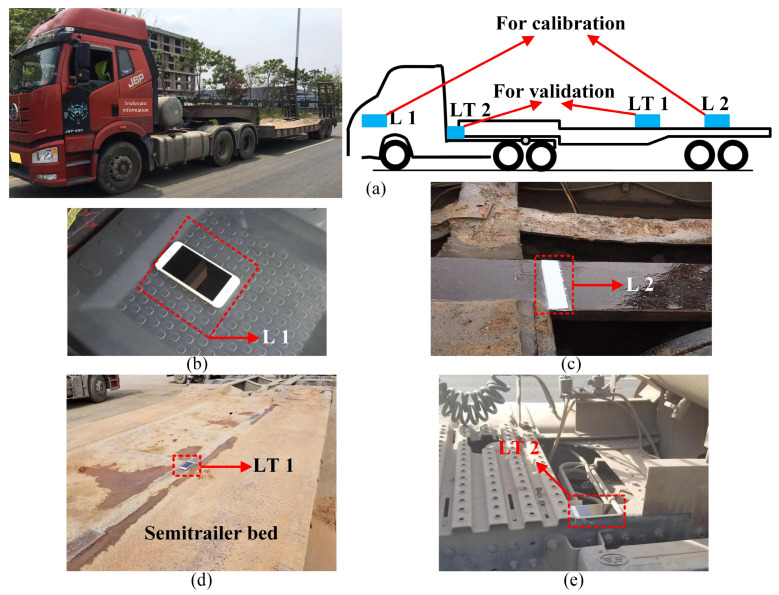
Sensor layout for tractor-semitrailer model calibration. (**a**) Sensor layout; (**b**) position of L1 for calibration; (**c**) position of L2 for calibration; (**d**) position of LT 1 for validation; (**e**) position of LT 2 for validation.

For practical use, a 5-axle tractor-semitrailer model was calibrated with the SHCM and DHCM. The test vehicle was an ordinary Jie-fang J6P tractor-semitrailer (Figure 12a), which maintained a constant taxiing speed of 2 m/s during the test. Figure 12a–c presents the layout of vehicle calibration sensors. Sensor L1 was installed above axle 1, and Sensor L2 was installed between axle 4 and axle 5. Information on the field measurements in the preparation phase is provided as follows: *b*_1_ + *b*_2_ = 3.46 m, *b*_1_ + *b*_3_ = 4.80 m, *b*_4_ + *b*_6_ = 8.50 m, *b*_5_ + *b*_6_ = 9.73 m, *b*_1_ + *b*_7_ = 3.75 m, the position of Sensor L1: *d*_5-axle,tractor_ = −0.29 m, and the position of Sensor L2: *d*_5-axle,semitrailer_ = 9.32 m. In addition, as shown in Figure 12d,e, Sensor LT 1 on the semitrailer (d_5-axle,semitrailer_ = 5.76 m) and Sensor LT 1 outside the tractor cab (d_5-axle,tractor_ = 2.36 m) were used to collect acceleration data and compare them with the simulated accelerations with the calibrated model to confirm the validity of the models calibrated with the DHCM. The validation is presented later in Section 4.

**Figure 13:**
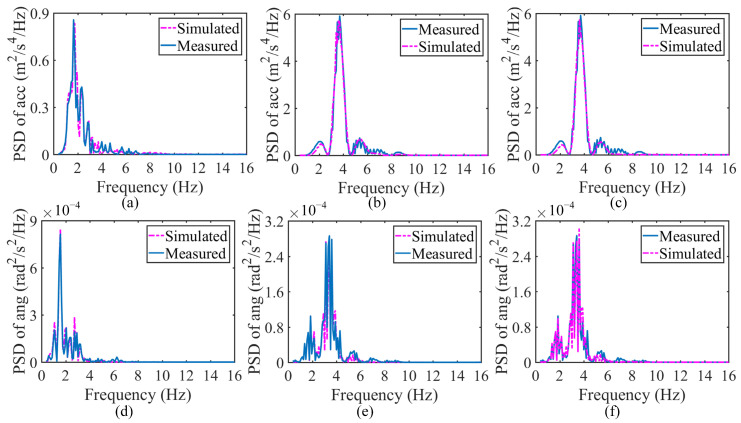
Comparisons for 5-axle vehicle calibration. (**a**) Accelerations at L1 by the SHCM; (**b**) accelerations at L2 by the SHCM; (**c**) accelerations by the DHCM; (**d**) angular velocities at L1 by the SHCM; (**e**) angular velocities at L2 by the SHCM; (**f**) angular velocities by the DHCM. Note: (**c**,**f**) only illustrate the comparisons in step 2 of the DHCM, since the comparisons in step 1 of the DHCM are the same as (**a**,**d**).

The comparisons of PSDs between simulated and measured responses at L1 and L2 for the SHCM are shown in Figure 13a,b,d,e, while the comparisons of PSDs between simulated and measured responses for the DHCM are shown in Figure 13c,f. All simulated vehicle responses agree with the measurements. The three calibrated 5-axle vehicle models generated from the identified parameters are labeled SHCM-1 (calibrated with the SHCM and responses at L1), SHCM-2 (calibrated with the SHCM and responses at L2), and DHCM-1&2 (calibrated with the DHCM and responses at L1 and L2). The parameters of the identified vehicle models are listed in Table A2.

### 3.4. Dynamic Characteristics of Calibrated Models

**Figure 14:**
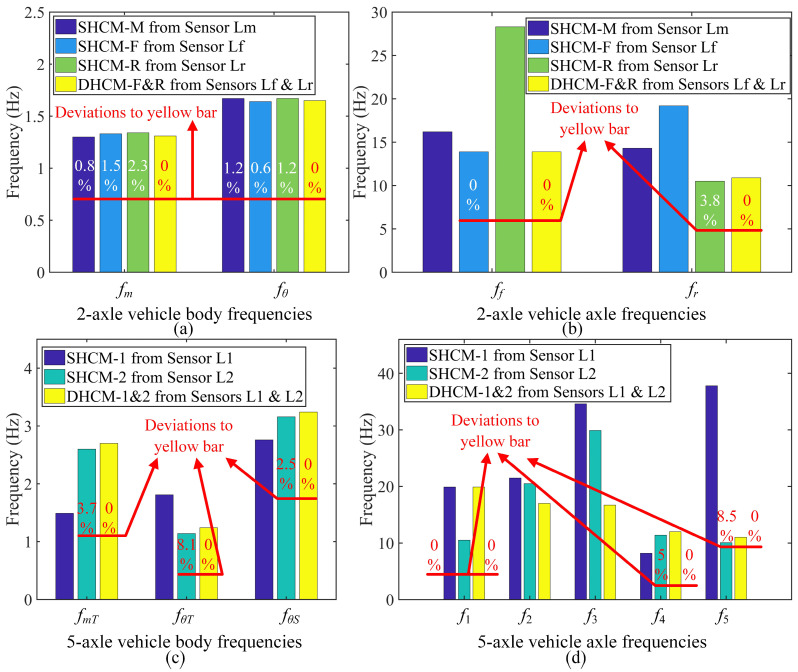
Natural frequencies of 2-axle and 5-axle vehicle models. (**a**) 2-axle vehicle bouncing and pitching frequencies; (**b**) 2-axle vehicle hop frequencies; (**c**) 5-axle vehicle bouncing and pitching frequencies; (**d**) 5-axle vehicle hop frequencies.

The natural frequencies (see Figure 14) corresponding to the bouncing, pitching, and axle hop motion modes of the 2-axle vehicle models and the 5-axle articulated vehicle models are calculated using Equation (29):

(29) $$\det\left(K-\mu M\right)=0$$

where *μ* = (2π*f*)^2^; *f* represents the natural frequencies of the vehicle systems. For simplicity, vehicle body frequencies refer to the natural frequencies corresponding to vehicle body motion modes, including bouncing and pitching; the same rule applies to the definition of vehicle axle frequencies, bouncing frequencies, pitching frequencies, et cetera.

For the calibrated 2-axle vehicle models SHCM-M, SHCM-F, SHCM-R, and DHCM-F&R, Figure 14a indicates minor differences in vehicle body frequencies, including bouncing frequencies *f_m_* and pitching frequencies *f_θ_*, between these models. In addition, Figure 14b reveals that the front axle hop frequency *f_f_* of SHCM-F is close to the *f_f_* of DHCM-F&R, as is the rear axle hop frequency *f_r_* of SHCM-R to *f_r_* of DHCM-F&R. These findings imply that the model calibrated with the DHCM contains detailed modal information about the motions of the vehicle body and axle components. In contrast, the model calibrated with the SHCM identifies only the vehicle body frequencies.

Likewise, for the calibrated 5-axle articulated vehicle models SHCM-1, SHCM-2, and DHCM-1&2, Figure 14c reveals that the vehicle body frequencies, including tractor bouncing frequencies *f_mT_*, tractor pitching frequencies *f_θT_*, and semitrailer pitching frequencies *f_θS_*, are similar between SHCM-2 and DHCM-1&2. However, the frequencies *f_mT_*, *f_θT_*, and *f_θT_* derived from SHCM-1 differ from the above frequencies. Therefore, relatively low robustness is shown when SHCM is used to calibrate the articulated vehicle model because the SHCM-1 model misidentifies the vehicle body motion modes. In addition, Figure 14d presents that the hop frequency *f*_1_ of SHCM-1 is close to *f*_1_ of DHCM-1&2, and that the hop frequencies *f*_4_ and *f*_5_ of SHCM-2 are close to *f*_4_ and *f*_5_ of DHCM-1&2. Overall, the model calibrated with the DHCM identifies vehicle body frequencies and most of the vehicle axle frequencies, while the models calibrated with the SHCM cover only a tiny part of this information.

## 4. Validation Results

The values of the calibrated parameters should be validated to approximate the actual values of the vehicle’s physical parameters. Since the actual values of the physical parameters cannot be determined directly, the principle of the validation process is to demonstrate the accuracy of the calibrated vehicle model, which consists of the calibrated parameters, compared to the realistic vehicle model. Furthermore, considering that the dynamic responses of the realistic vehicle model can be measured directly, the accuracy is demonstrated by the agreement of the simulated acceleration generated by the calibrated vehicle model with the measured acceleration.

### 4.1. Principle of Acceleration Simulation

The vertical acceleration responses can be simulated by substituting the input road profiles into the equations of motion of the models calibrated with SHCM and DHCM. Thus, the prerequisite for the acceleration simulation is obtaining the modeled profiles.

The vertical displacements of road roughness are condensed into an idealized form of PSDs and divided into A-H grades according to ISO 8608 [41]. The expression for the PSD, i.e., G*q*(*n*), is given by:

(30) $$G_{q}\left(n\right)=G_{q}(n_{0}){\left(n/n_{0}\right)}^{-w}$$

where *n* is the spatial frequency; *n*_0_ = 0.1 cycles/m is the reference spatial frequency; G*q*(*n*_0_) is the PSD for the reference spatial frequency and given in ISO 8608; *w* = 2 is the exponent of the PSD under non-logarithmic coordinates. The basic idea of road modeling is to obtain the time history of road profiles from the idealized PSD. In practice, road profiles have been numerically modeled by Godbole et al. [2], Wang et al. [14], and Lin et al. [37].

With the modeled road profiles at the vehicle axles as input in Equation (1) of the calibrated vehicle models, the acceleration responses at the COGs of the vehicle bodies can be simulated. The acceleration response at any position of a 2-axle vehicle then results from Equation (3); the acceleration response at any position of the tractor and semitrailer of a 5-axle vehicle is obtained from Equation (15) and Equation (17), respectively.

### 4.2. Comparison between PSDs of Measured and Simulated Acceleration

#### 4.2.1. Measured Acceleration PSD for the 2-Axle Vehicle

**Figure 15:**
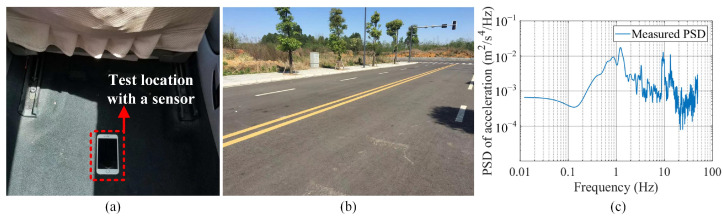
The 2-axle vehicle test to obtain measured PSD. (**a**) test location for acceleration acquisition; (**b**) class B road; (**c**) measured PSD.

A field test, referred to as Case 1-1, was conducted to record the acceleration response of the 2-axle sedan under the natural driving condition. The test vehicle was the sedan used for the model calibration in Section 3.2, driven at a constant speed of 10 m/s on a class B road, as shown in Figure 15b. The test location for acceleration response acquisition was set at 1.7 m from the front axle of the vehicle, where a smartphone was installed for data acquisition (Figure 15a). Figure 15c presents the sensor data that were converted to PSD of the measured acceleration.

#### 4.2.2. Comparison of PSDs for the 2-Axle Vehicle

**Figure 16:**
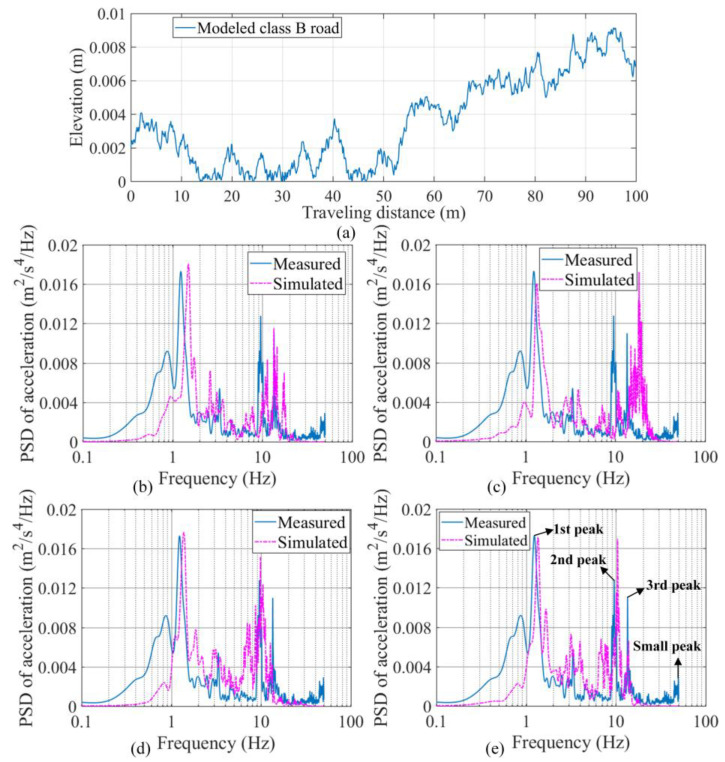
Comparisons for the sedan. (**a**) Modeled road; (**b**) PSD by SHCM-M; (**c**) PSD by SHCM-F; (**d**) PSD by SHCM-R; (**e**) PSD by DHCM-F&R.

In accordance with the road condition in the field test Case 1-1, a modeled profile of the class B road is presented in Figure 16a. With the road profile input, the simulated accelerations at the test location in Case 1-1 (Figure 15a) were obtained for four calibrated vehicle models (SHCM-M, SHCM-F, SHCM-R, and DHCM-F&R) by solving the corresponding equation of motion. The simulated accelerations were then compared with the measured acceleration (Figure 15c) in the frequency domain.

As shown in Figure 16e, the measured PSD curve contains three primary peaks and one secondary peak. As shown in Figure 16b–e, in the frequency range of 0.1–10 Hz, both the first peak value and the corresponding frequency of the measured PSD agree with the values and frequencies of the PSDs calculated by SHCM-M, SHCM-F, SHCM-R, and DHCM-F&R. Since the first peak value of the measured PSD is more significant than the others, the PSDs simulated by the above models all present some degree of similarity to the measured PSD. In the 10–20 Hz range, the PSD simulated by DHCM-F&R exhibits a high degree of similarity, as the frequencies of the second and third peaks are close to those of the measured PSD. 

Furthermore, in the range of 40–50 Hz, a small peak in the measured PSD does not match the peaks in any of the simulated PSDs. This discrepancy between the simulated and measured PSDs could be due to (1) the unreliable accuracy of smartphone sensors in the frequency range beyond 40 Hz [31], (2) the mismatch between modeled and real road profiles, and (3) the use of planar and linear vehicle models.

#### 4.2.3. Measured Acceleration PSD for the Articulated Vehicle

**Figure 17:**
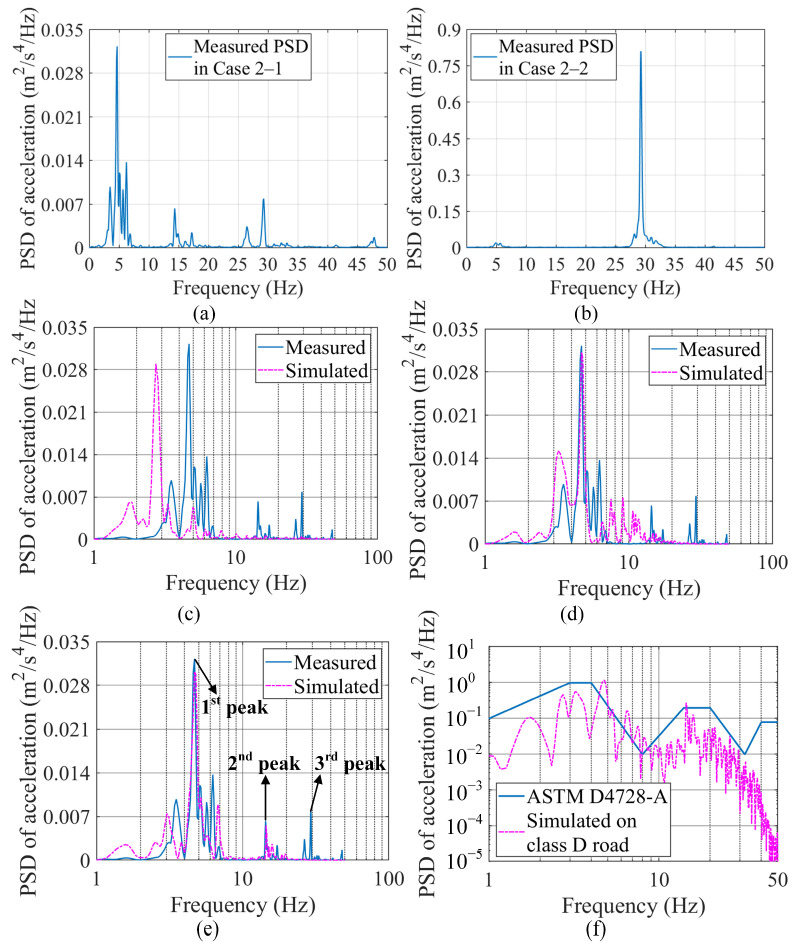
Comparisons for the tractor-semitrailer. (**a**) Measured PSD in Case 2-1; (**b**) measured PSD in Case 2-2; (**c**) PSD by SHCM-1; (**d**) PSD by SHCM-2; (**e**) PSD by DHCM-1&2. (**f**) comparison between PSD by DHCM-1&2 and PSD from standard.

Cases 2-1 and 2-2 represented the field tests for the 5-axle tractor-semitrailer vehicle. Case 2-1 was conducted to identify the acceleration response, while Case 2-2 was conducted to identify vibration sources other than vehicle motions. As shown in Figure 12d,e, Sensor LT 1 in Case 2-1 was installed on the semitrailer (*d*_5-axle,semitrailer_ = 5.76 m) to collect acceleration data; Sensor LT 2 in Case 2-2 was installed outside the tractor cab (*d*_5-axle,tractor_ = 2.36 m) to collect other vibration data. The test tractor-semitrailer was driven at 3 m/s on the same road as in Case 1-1. Figure 16a presents the modeled profile of such a road. The PSD of the measured acceleration of the 5-axle vehicle was obtained from the data of Sensor LT 1 (sampling at 100 Hz) in Case 2-1, as shown in Figure 17a.

#### 4.2.4. Comparison of PSDs for the Articulated Vehicle

Figure 17c–e presents the PSDs of the simulated accelerations in Case 2-1 by three calibrated vehicle models (SHCM-1, SHCM-2, and DHCM-1&2). The simulated PSDs are then compared with the measured PSD, which contains three primary peaks. In the frequency range of 1–10 Hz, the first peak value and the corresponding frequency of the measured PSD agree with the values and frequencies of the PSDs calculated by SHCM-2 and DHCM-1&2, while the above values differ from those derived from SHCM-1. In the 10–20 Hz range, a high degree of agreement between the PSD simulated by DHCM-1&2 and the measured PSD is evident, as the frequency of the second peak, with a value of 15 Hz, is close to its counterpart in the measured PSD. In the range of 25–30 Hz, the third peak in the measured PSD does not correspond to the peaks in any of the simulated PSDs. A further comparison of the measured PSDs between Figure 17a,b indicates that this vibration did not originate from the motions of the vehicle, but was possibly caused by a component of the tractor (e.g., the engine) and decayed as it propagated to the semitrailer. These findings demonstrate that the simulated acceleration by the DHCM-identified model possesses higher precision than those by the SHCM-identified models.

In addition, the highly generalized acceleration PSDs of the ASTM standard [42] should reveal some characteristics of the measured PSD of the 5-axle vehicle, since the standard PSDs were derived from accelerations of all types of articulated vehicles, including the type in this work. As can be seen in Figure 17f, the two high peaks of the standard PSD correspond to those of the simulated PSD of the 5-axle vehicle model (calibrated with the DHCM) driving on a class D road. To some extent, the similar configuration of the standard and simulated PSDs confirms the precision of the simulation.

### 4.3. Comparison of RMS Values

Regardless of vehicle type, the comparisons between the simulated and measured PSDs contributed to inferring the high precision of the models calibrated with the DHCM compared to those calibrated with the SHCM. However, the extent of this precision improvement needs to be further quantified.

**Figure 18:**
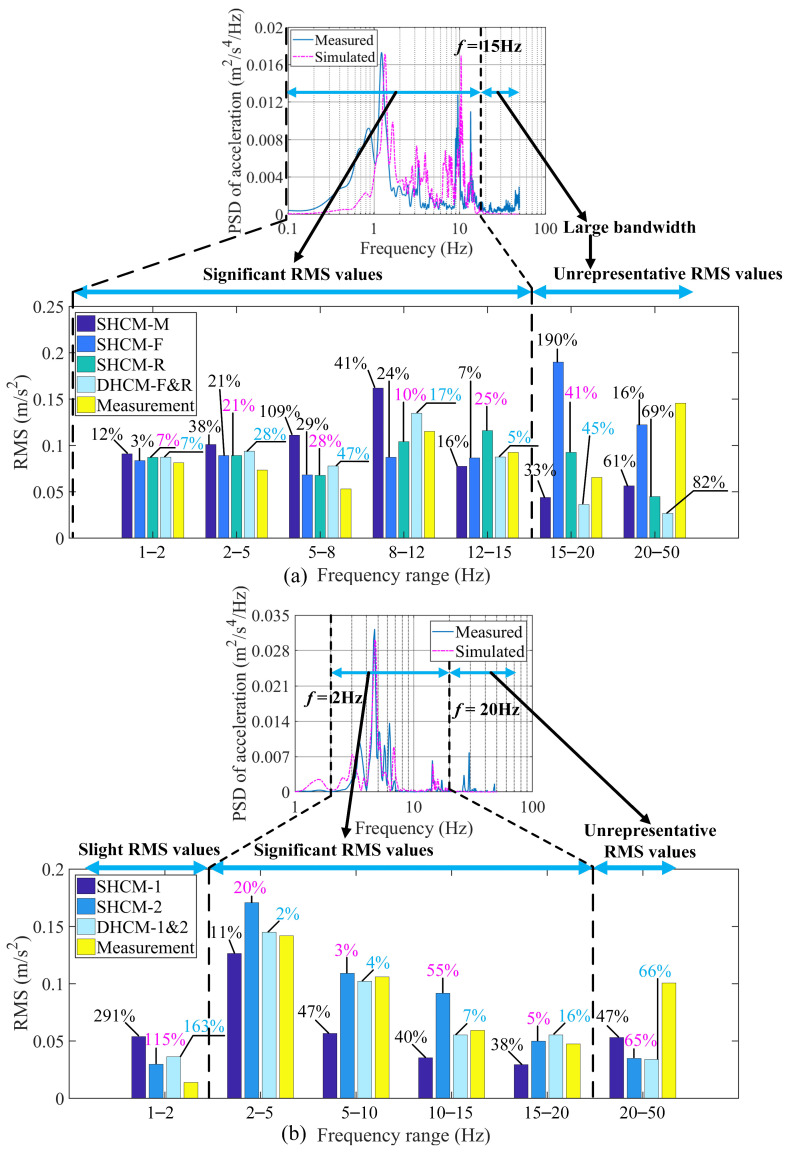
RMS values and relative RMS errors. (**a**) For the 2-axle vehicle; (**b**) for the 5-axle vehicle.

The RMS acceleration value of a PSD curve under a certain frequency segment quantitatively evaluates the dynamic effect of the acceleration response within this frequency range. Therefore, the relative RMS error between the simulated PSD curve and the measured PSD curve quantifies the differences between the two PSDs in a given frequency range. The smaller the relative RMS error, the more similar the two PSD curves are. Concretely, the RMS value within a range from *f_i_* to *f_j_* is calculated as follows:

(31) $$RMS=\sqrt{PSD_{\mathrm{acc}}\left(f_{i}\right)\frac{BW}{2N}+\sum_{k=i+1}^{j-1}PSD_{\mathrm{acc}}\left(f_{k}\right)\frac{BW}{N}+PSD_{\mathrm{acc}}\left(f_{j}\right)\frac{BW}{2N}}$$

where *PSD*_acc_(*f*) is the PSD value of the acceleration corresponding to frequency *f*; *BW* is the bandwidth of the entire frequency range; *N* is the number of samples. The RMS values within different frequency segments derived from the vehicle models and measurements are shown in Figure 18 (which provides a better understanding of the differences between various models), where the relative RMS errors are quantified as follows:

(32) $$RE=\left|RMS_{\mathrm{simulated}}-RMS_{\mathrm{measured}}\right|/RMS_{\mathrm{measured}}$$

The frequency range of PSDs for 2-axle vehicle models was divided into seven segments: 0.1–2, 2–5, 5–8, 8–12, 12–15, 15–20, and 20–50 Hz to calculate the RMS values separately. Since the low PSD values within 15–50 Hz were not representative of the overall PSD shape and thus the relative RMS errors in this frequency range were meaningless, the comparisons of RMS values were performed in the frequency range of 0.1–15 Hz. Similarly, the comparisons of the RMS values for the 5-axle vehicle models were performed in the 2–20 Hz range, where the RMS values were significant.

As Figure 18a displays, in the frequency range of 0.1–15 Hz, there are consistencies with the RMS values from the 2-axle vehicle models (SHCM-R and DHCM-F&R) and the RMS values from the measurement. Moreover, the accuracy of the RMS values from DHCM-F&R (*RE* = 5%) is better than that from SHCM-R (*RE* = 25%) only in the range of 12–15 Hz. Accordingly, a limited improvement in accuracy is observed for the 2-axle vehicle model calibrated with DHCM. The deeper reason for this observation is that 2-axle vehicles are more simply configured and have shorter bodies than 5-axle vehicles, so the SHCM fulfills the calibration requirements for 2-axle vehicles to a certain extent and adding another sensor in the calibration process is not necessary. By using the DHCM, only the vehicle axle parameters of 2-axle vehicles were better identified. This point is reflected in the improved accuracy of the simulated PSD curves in the range of 12–15 Hz, which corresponds to the frequencies of the vehicle axles. In contrast (Figure 18b), in the frequency range of 2–20 Hz, only between the RMS values derived from DHCM-1&2 and the RMS values from the measurement are good consistencies with the RMS values presented, indicating that a 5-axle vehicle model can be calibrated better with the DHCM than with the SHCM.

In addition, the frequency range below 20 Hz is the focus of multidisciplinary research because the highest vibration energies of transported objects were concentrated in the low-frequency range [4,5,6,7,8,10], and the principle natural frequencies of bridges in vehicle–bridge interaction dynamics research were usually below 20 Hz [13,14,15,16,17,18,19,20,21,22]. Therefore, the simulated dynamic responses, i.e., accelerations for cargo damage analysis and vehicle tire reaction forces for bridge property identification, are valuable in this range. As shown in Figure 18b, the relative RMS errors derived from DHCM-1&2 are less than 16% within 2–20 Hz, which demonstrates the authenticity and reliability of the simulated dynamic response of the articulated vehicle model calibrated with DHCM.

## 5. Conclusions and Discussions

This work aimed to develop a practical calibration method for identifying unladen and laden multi-axle articulated vehicle models using an optimal arrangement of sensors. Accordingly, a novel DHCM method was proposed based on parameter sensitivity analysis and MPGA. For practical application, the DHCM and the existing SHCM were used to calibrate 2-axle sedan and 5-axle tractor-semitrailer models. Experimental validation was performed to demonstrate the higher accuracy of the models calibrated with the DHCM over those calibrated with the SHCM by comparing the measured and simulated acceleration PSDs on the vehicle bodies.

### 5.1. Conclusions

The conclusions are presented below.

1. The existing SHCM is suitable for calibrating small-sized vehicle models but not for multi-axle articulated vehicle models.
2. Parameter sensitivity analysis of a 5-axle tractor-semitrailer model reveals that the vehicle models should be calibrated using two sensors installed on the front and rear articulated parts of the vehicle.
3. Based on the optimal sensor arrangement, the DHCM can be conveniently implemented using smartphones to record the vehicle responses and an MPGA to determine the vehicle parameters.
4. The DHCM, supplemented by analytical methods and the FEM, is suitable for the identification of laden articulated vehicle models and specific vehicle parameters, including the height of the laden semitrailer’s COG.
5. Regardless of vehicle type, the measured PSD agrees better with the simulated PSD from models calibrated with the DHCM than with that from models calibrated with the SHCM, indicating that the DHCM is suitable for calibrating small-sized vehicle models and articulated vehicle models.
6. Relative RMS errors from the articulated vehicle model calibrated with the DHCM are below 16% within 2–20 Hz, demonstrating that this model realistically simulates the dynamic response of an articulated vehicle in the low-frequency range.

### 5.2. Discussions

The requirements and limitations of the DHCM are explained below:

1. The test vehicle should be driven on a flat road during the DHCM. Class A-B roads are therefore recommended.
2. Further work could focus on integrating the DHCM into low-cost hardware.

In addition, there are two ways to improve the calibration results of the DHCM. One is to use sensors with more reliable accuracy in the high-frequency range instead of smartphone sensors. The other is to use more sophisticated vehicle models, e.g., taking into account the configurations of the suspended cab and tandem axles in the models.

## Data Availability

The data used to support the research of this paper are available from the corresponding author upon reasonable request.

## Appendix A

**Table A1:** Normalized parameters of the 2-axle sedan vehicle model.

Sensor Locations	Methods	Normalized Parameters (*L_f_*: m)									
		*L_f_*	*m_fn_*	*m_rn_*	*I_Zn_*	*k_fn_*	*k_rn_*	*c_fn_*	*c_rn_*	*k_tfn_*	*k_trn_*
Lm	SHCM	1.43	0.07	0.08	1.78	32.42	58.94	1.85	2.84	642.66	606.71
Lf	SHCM	1.07	0.07	0.06	1.42	44.68	34.00	2.34	2.09	481.17	879.74
Lr	SHCM	1.38	0.02	0.15	1.66	35.68	53.64	1.47	3.39	589.45	594.84
Lf and Lr	DHCM	1.18	0.06	0.10	1.76	41.67	47.77	2.18	2.97	448.76	408.65

**Table A2:** Normalized parameters of the 5-axle articulated vehicle model.

Sensor Locations	Methods	Normalized Parameters (Geometric Parameters: m)								
		*m_Sn_*	*m*_1*n*_	*m*_2*n*_	*m*_3*n*_	*m*_4*n*_	*m*_5*n*_	*I_Tn_*	*I_Sn_*	*k*_1*n*_
L1	SHCM	2.34	0.03	0.03	0.03	0.22	0.03	2.29	26.96	60.87
L2	SHCM	2.50	0.30	0.16	0.06	0.40	0.46	44.79	53.29	150.93
L1 and L2	DHCM	4.77	0.11	0.40	0.48	0.57	0.34	45.75	95.89	198.83
		*k*_2*n*_	*k*_3*n*_	*k*_4*n*_	*k*_5*n*_	*c*_1*n*_	*c*_2*n*_	*c*_3*n*_	*c*_4*n*_	*c*_5*n*_
L1	SHCM	90.21	27.13	25.19	484.11	3.85	3.01	4.46	7.67	6.40
L2	SHCM	40.47	582.96	309.91	441.12	2.61	17.60	7.95	20.57	4.58
L1 and L2	DHCM	93.02	869.88	537.26	1313.27	12.57	33.04	6.80	36.52	23.60
		*k_t_*_1*n*_	*k_t_*_2*n*_	*k_t_*_3*n*_	*k_t_*_4*n*_	*k_t_*_5*n*_	*a*_1_	*a*_2_	*b*_1_	*b*_6_
L1	SHCM	447.09	499.58	1371.08	573.80	1331.17	0.43	0.84	1.24	6.51
L2	SHCM	1170.41	2557.61	1576.68	1610.16	646.11	0.96	0.75	1.91	4.37
L1 and L2	DHCM	1460.53	4488.70	4360.85	2689.24	808.00	0.97	0.82	1.32	4.14
